# Hope Hospital

**Published:** 1909-09-25

**Authors:** J. H. Taylor

**Affiliations:** Hon. Sec. Salford Div. B.M.A. Holly House, 299 Eccles New Road, Salford


### HOPE HOSPITAL.

Sir,—I have to thank you for copy of your issue of The Hospital for September 11, in which on page 625 you refer to me in connection with Hope Hospital. Towards the end of your article you quote Mr. Bretherton as saying, "When a shortage of staff was pointed out to us by the Local Government Board the Guardians remedied it immediately." This must refer to some ancient history, for there has not been any addition to the medical staff for a long time. Further, Dr. McVail in his report to the Royal Commission on the Poor Laws said the place was so understaffed that the "work was bound to be rushed." A few weeks ago Mr. Lowry (the Local Government Board inspector) drew the Guardians' attention to this, and urged them to attend to it as soon as possible. So far from doing so are they that Mr. Bretherton now actually denies that there is any understaffing (see your own article). In other words, he denies the correctness of Dr. McVail's report and the Local Government Board inspector's opinion. You may judge for yourself when I say that there are only two resident medical officers, who are responsible for attending to between 800 and 900 patients.

I will only add that the *Lancet* and the *British Medical Journal* have both refused to insert the Guardians' latest advertisement and have put the post on the "Warning Notice" pages.

Yours faithfully,
J. H. Taylor, M.A., M.B.,
Hon. Sec. Salford Div. B.M.A.

Holly House, 299 Eccles New Road, Salford,
September 21, 1909.

